# *Drosophila* Importin Alpha 1 (Dα1) Is Required to Maintain Germline Stem Cells in the Testis Niche

**DOI:** 10.3390/cells13060494

**Published:** 2024-03-12

**Authors:** James Heaney, Jiamin Zhao, Franca Casagranda, Kate L. Loveland, Nicole A. Siddall, Gary R. Hime

**Affiliations:** 1 Department of Anatomy and Physiology, University of Melbourne, Parkville, VIC 3010, Australia; 2 Centre for Reproductive Health, Hudson Institute of Medical Research, Clayton, VIC 3168, Australia; 3 Department of Molecular and Translational Sciences, Monash University, Clayton, VIC 3800, Australia

**Keywords:** *Drosophila*, spermatogenesis, importin

## Abstract

Stem cell maintenance and differentiation can be regulated via the differential activity of transcription factors within stem cells and their progeny. For these factors to be active, they need to be transported from their site of synthesis in the cytoplasm into the nucleus. A tissue-specific requirement for factors involved in nuclear importation is a potential mechanism to regulate stem cell differentiation. We have undertaken a characterization of male sterile importin alpha 1 (*Dα1*) null alleles in *Drosophila* and found that Dα1 is required for maintaining germline stem cells (GSCs) in the testis niche. The loss of GSCs can be rescued by ectopic expression of Dα1 within the germline but the animals are still infertile, indicating a second role for Dα1 in spermatogenesis. Expression of a Dα1 dominant negative transgene in GSCs confirmed a functional requirement for Dα1 in GSC maintenance but expression of the transgene in differentiating spermatogonia did not exhibit a phenotype indicating a specific role for Dα1 within GSCs. Our data indicate that Dα1 is utilized as a regulatory protein within GSCs to facilitate nuclear importation of proteins that maintain the stem cell pool.

## 1. Introduction

Transport of proteins and RNA molecules across a nuclear membrane that separates the nucleus from the cell cytoplasm is an essential function of all eukaryotes. In development, cellular differentiation is dependent upon the efficient transportation of proteins that are synthesized in the cytoplasm but are required in the nucleus to interact with DNA. These can include transcription factors, histones and cell cycle regulators, all of which are important for the regulation of cell fate dynamics (reviewed in [1,2]). Karyopherins, a multigene family of nuclear transport receptors, mediate the transport of large macromolecules (cargo) across the nuclear pore complexes (NPCs) into the nucleus (importins) or out of the nucleus (exportins) (reviewed in [3,4]).

The purported classical nuclear import pathway asserts that cargo greater than about 50 kDa containing a classical nuclear localisation signal (cNLS) binds to a specific Importin α protein, which functions as an adaptor to link cNLS-cargos to Importin β1 [5,6]. This complex then passes through the NPC by binding phenylalanine-glycine (FG) repeats present in many nucleoporins. Following translocation into the nucleus, the importin-cargo complex dissociates in the presence of high RAN-GTP and the free importin proteins are recycled back to the cytoplasm (reviewed in [3,7]).

Selective transport of cargo such as transcription factors is possible due to the capacity of individual importin αs to preferentially bind different cargoes [8,9]. Vertebrate Importin αs are expressed in distinct cell- and tissue-specific patterns, further enabling the nuanced control of nuclear import [10,11,12]. Importin α family members are divided into three clades: Importin α1, α2 and α3. The human genome encodes seven *importin* αs (three α1s, and two of both α2s and α3s), while the mouse genome encodes six *importin* αs (two α1s, α2s and α3s). *Drosophila melanogaster* is an excellent model organism in which to study the role of Importin α proteins in development since the *Drosophila* genome only encodes four *importin αs* (a single *Dα1*, *Dα2* and *Dα3* and a more divergent importin *αKap4*). Dα1, Dα2 and Dα3 contain conserved Importin β1 and cNLS-binding domains [13,14,15,16] and, like vertebrate Importins, have differential stage- and cell-specific expression patterns [16,17,18,19,20].

The differential expression and functional requirement for Importins in spermatogenesis and oogenesis across many species, including *Drosophila*, has previously been described [2,20,21,22,23,24,25,26,27]. In flies, mutations in *importin α3* (*Dα3*) result in larval lethality [24]. Conversely, *importin α1 and α2* (*Dα1* and *Dα2*) mutants are viable as adults, but small deficiencies that cause null mutations in either of these genes result in severe defects in gametogenesis, with females and males both being sterile [24,26]. In *Dα2* mutants, male sterility was found to be caused by sperm individualisation defects in late spermatogenesis [24], while in females, *Dα2* was found to be required for the assembly of ring canals in oogenesis [26]. The precise cause of sterility in *Dα1* remains unknown [28].

The functional requirement for Dα1 in *Drosophila* gametogenesis was originally discovered from experiments using a small deficiency (*Df(3L)α1S1*) that causes partial or full deletion of seven genes, including *Dα1* [28]. *Df(3L)α1S1* homozygous flies are viable to adulthood but are sterile. Both male and female sterility could be rescued by driving the expression of a UAS-Dα1 transgene driven by an *Act5CGal4* driver, thus confirming a function for Importin α1 in fertility [28]. Interestingly, driving expression of UAS-Dα2 and UAS-Dα3 transgenes in *Dα1* homozygous males and females did not rescue sterility, thus revealing a paralogue-specific function for *Dα1* in both spermatogenesis and oogenesis [28].

While the precise mechanisms that cause male sterility in *Dα1* homozygous flies remain uncharacterised, testes dissected from these animals have been reported as being smaller in size, and spermatocytes appear morphologically aberrant [28]. Despite these findings, a potential function for Dα1 in early spermatogenesis, including stem cell function, has remained unexamined. In this study, we show that Dα1 is required in testes for spermatogonial germline stem cell (GSC) maintenance. We show that *Dα1* mutant GSCs are not maintained in the niche and are lost as a result of premature differentiation. Our findings highlight the importance of specific proteins for controlling nuclear entry at the earliest stages of spermatogenesis.

## 2. Materials and Methods

### 2.1. Fly Strains

All flies were raised on standard molasses-based food at 18 °C, 25 °C or 29 °C, as specified in the text. Flies were primarily dissected at 0 days (Newly Emerged) and 7 days post-eclosion unless otherwise stated. Fly stocks used in this study obtained from the Bloomington *Drosophila* Stock Centre (BDSC, Bloomington, IN, USA) include *w^1118^*, *nanos*-*Gal4* and *bam*-*Gal4*. *Df(3L)α1S1* was provided by Robert Fleming (Trinity College, Hartford, CT, USA), *Dα1^Z5234^* and *Dα1^Z1703^* (from the Zuker male sterile collection) were obtained from Barbara Wakimoto (University of Washington, Seattle, WA, USA). *nanos*-*Gal4* was combined with *UAS-Gal4* (BDSC, Bloomington, IN, USA) to amplify Gal4 expression. The generation of UAS-*Dα1^DN^* transgenic flies is described below.

### 2.2. Generation of UAS-Dα1^DN^ Transgenic Flies

Primer sequences were designed to allow for a deletion of 85 amino acids from the N-terminus of the full-length Dα1 protein—5′-GCTGGAAGCGGGGCACAGCC C-3′ and 5′-GCTAAAAGTTAAATCCGGTGCTGGGCATGTTGTCC-3′. The *Dα1* truncated cDNA sequence was amplified from cDNA provided by the Goldfarb lab, ligated into a pTMW expression vector as per manufacturer’s instructions, using pENTR^TM^ as an intermediate cloning step. Plasmids were sent to BestGene Inc. (Chino Hills, CA, USA) for generation of transgenic flies. The Myc tag in the pTMW expression vector allows for confirmation of the expression of the construct in all experiments using the 9E10 antibody.

### 2.3. Immunostaining and Image Analysis

**Figure 1:**
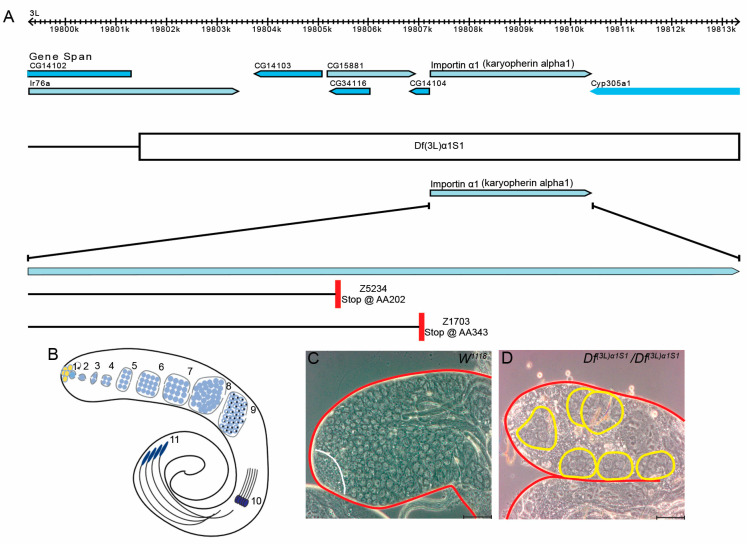
Loss of *Dα1* gene function results in defects in spermatogenesis. (**A**) Image of *Dα1* and surrounding genes modified from Flybase. Deficiency *Df(3L)α1S1* deletes a region of the left arm on the 3rd chromosome approximately 12 kb in length and covers *Dα1* and 6 other genes. The *Z5234* loss of function allele is caused by a point mutation resulting in a premature stop codon at amino acid 202, while the *Z1703* loss of function allele is caused by a point mutation resulting in a premature stop codon at amino acid 343; (**B**) Schematic depiction of *Drosophila* spermatogenesis. Germline stem cells (1) surround somatic hub cells (yellow). A pair of Cyst progenitor cells (grey) surround the GSC. Circular arrow depicts the self-renewal capacity of the GSC. Gonialblasts (2), progeny of GSCs, are surrounded by a pair of differentiated somatic cyst cells (grey). Gonialblasts undergo four rounds of mitosis (3–6). Spermatogonia differentiate into spermatocytes (7) and undergo meiosis (8–9) to produce 64 round spermatids (9). (10) shows early elongating spermatids (only 4 depicted), (11) shows 4 elongating spermatids; (**C**,**D**) phase contrast microscopy shows the tip of the *Drosophila* testes. Testes have been outlined in red. (**C**) A testis dissected from a *w^1118^* newly eclosed adult shows the presence of germline cells at all stages of early spermatogenesis, including small undifferentiated germline stem cells and gonialblasts to differentiating spermatogonia and spermatocytes (the stem cell niche that includes stem cells, gonialblasts and spermatogonia is indicated by the white line); (**D**) Homozygous *Df(3L)α1S1* testes lack young germ cells. Large germline cysts containing fewer than 16 cells are present in the apex of the homozygous *Df(3L)α1S1* testis (outlined in yellow). Cells within the cyst appear to be spermatocytes. Scale Bars, 5 μm.

Appropriately aged males (day of eclosion (hatching) or 7 days post-eclosion) were dissected in PBS, fixed for 15 min in 4% Formaldehyde in PBT (PBS + 0.2% Triton X-100 (Sigma-Aldrich, Burlington, MA, USA)), washed for 3 × 5 min in PBT, and then blocked for 30 min in PBTH (5% Normal Horse Serum in PBT) using an agitator. Testes were then incubated overnight at 4 °C with primary antibodies diluted in PBTH. Samples were washed a further 3 × 5 min in PBT before secondary antibody incubation was conducted for 2 h on an agitator at room temperature in PBTH, protected from light. Samples were washed for a further 3 × 5 min in PBT before testes were mounted on slides in Prolong™ Gold Antifade Reagent with Dapi (Invitrogen, Waltham, MA, USA). Antibodies used in this study include 1:100 rat anti-(D)E-Cadherin (Developmental Studies Hybridoma Bank, University of Iowa, Iowa City, IA, USA (DSHB—catalogue number #DCAD2)), 1:100 goat anti-Vasa (Santa Cruz Biotechnology, Dallas, TX, USA #dc-13), 1:1000 Rabbit anti-Dα1 (Gift of Bernard Mechler and Istvan Kiss, Biological Research Center of the Hungarian Academy of Science, Szeged, Hungary), and 1:100 Mouse anti-Myc 9E10 (DSHB #9E 10). Secondary antibodies (Donkey anti-Mouse Alexa Fluor 488/594/647 (#A-21202/A-21203/A-31571), Donkey anti-Goat Alexa Fluor 488/594/647 (#A-11055/A-11058/A-21447) and Donkey anti-Rat Alexa Fluor 488/594/647 (#A-21208/A-21209/A78947)) (obtained from Thermo Fisher Scientific, Scoresby, VIC, Australia) were used at a dilution of 1:500. Images were acquired using a Zeiss LSM510 Confocal Microscopes as serial optical sections (z-stacks) optimized to acquire overlapping sections. FIJI/ImageJ, version win64 was then used to process images and add scale bars. Adobe photoshop version 22.2 was used to compile the figure panels. We used FlyBase (release 6.14) to obtain image data in Figure 1A.

### 2.4. Phase Contrast Microscopy

Testes were dissected in Ringers solution and 3–4 individual testes were immediately mounted under a glass coverslip in Ringers solution. The slide was then imaged immediately as the testes flatten under the pressure of a coverslip and removal of liquid by lateral flow. Testes were imaged using a Zeiss Axioskop2 Plus microscope (Carl Zeiss Pty Ltd., Macquarie Park, NSW, Australia). The images were taken using a 20X objective.

### 2.5. Statistical Analysis and Creation of Graphs

GraphPad Prism version 7.01 was used to analyse data and prepare graphs, significant difference was calculated using the students *t*-test. Results were considered significant if *p* < 0.05. Error bars on all graphs represent the standard error of the mean.

## 3. Results

### 3.1. Mutations in Dα1 Cause a Loss of Spermatogonial Germline Stem Cells

The effects of *Dα1* mutations on later stages of *Drosophila* spermatogenesis have previously been described [28]; however, no reports have detailed a functional requirement for Dα1 in the regulation or maintenance of germline stem cells (GSCs). To investigate this, we dissected and analysed testes from homozygous *Df(3L)α1S1* flies, which carry a small chromosomal deletion covering the *Dα1* gene and six neighbouring genes (Figure 1A).

Spermatogenesis in *Drosophila* adults is well described (Figure 1B; reviewed in [29,30]). GSCs reside at the apical tip of a tubular testis, and these stem cells asymmetrically divide to self-renew and produce differentiated daughter cells called gonialblasts (GB). In a process similar to mammalian spermatogenesis, each gonialblast undergoes four rounds of mitosis with incomplete cytokinesis to generate a cyst of 16 primary spermatocytes. Spermatocytes, after growth, undergo meiosis with incomplete cytokinesis to form a cyst of 64 interconnected haploid spermatids, which are then converted into mature sperm in a process known as spermiogenesis. Previous reports have attributed male sterility in homozygous *Df(3L)α1S1* flies to loss of *Dα1* since expression of a UAS-Dα1 transgene driven by an *Act5CGal4* driver could rescue sterility [28]. To further characterise the cause of male sterility, we dissected testes from *Df(3L)α1S1* mutant flies and examined the cellular morphology of the testes using phase contrast microscopy. Testes dissected from homozygous *Df(3L)α1S1* flies appeared aberrant in morphology, lacking early germ cells which are normally easily identifiable under phase contrast microscopy (Figure 1C,D). Additionally, atypical cysts of less than 16 large germ cells resembling spermatocytes were frequently observed at the apical tip of mutant testes, suggestive of early spermatogenesis defects (Figure 1D), which are not observed in wildtype testes (Figure 1C).

**Figure 2:**
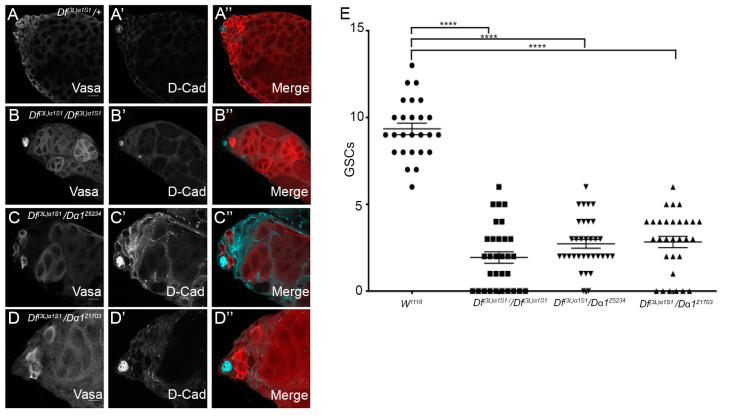
GSCs are lost in *Dα1* mutants. (**A**–**A”**) Representative confocal micrographs of testes dissected from 0 day old *Df(3L)α1S1/+* heterozygous control fly; (**B**–**B”**) *Df(3L)α1S1/Df(3L)α1S1* homozygous mutant; (**C**–**C”**); *Df(3L)α1S1/Dα1^z5324^* transheterozygote; and (**D**–**D”**) *Df(3L)α1S1/Dα1^z1703^* transheterozygote. (**B**–**D”**) Vasa (germ cells) and D-cad (hub and cyst cell membranes) labelling show a loss of germ cells from all mutants. The presence of germline cysts containing less than the full complement of large germ cells (likely spermatocytes) was observed in some testes close to the hub. (**E**) Scatterplot and statistical analysis showing significant loss of germline stem cells (GSC) when comparing the number of germline stem cells of adult *w^1118^* (control) testes to adult *Dα1* mutant testes. The average number of GSCs in tested dissected from *w^1118^* (9.35 (±1.67)) is significantly more that the average number of GSCs counted in *Df(3L)α1S1* homozygote testes (1.94 (±1.82)) GSCs, *Df(3L)α1S1/Dα1^Z5234^* transheterozygous testes (2.727 (±1.46)) GSCs and *Df(3L)α1S1/Dα1^Z1703^* transheterozygous testes (2.83 (±1.76)) GSCs. **** *p* < 0.0001. Scale Bars, 20 μm.

We could not rule out the possibility that the morphological defects observed in homozygous *Df(3L)α1S1* mutant testes were due to a loss of function of any of the six other genes affected by the chromosomal deletion. To explore the impact of Dα1 loss on the initial stages of spermatogenesis, we bred two *Drosophila* strains harbouring point mutations in the *Dα1* gene (Figure 1A), previously induced by ethyl methanesulfonate (EMS) treatment [31], with *Df(3L)α1S1* mutants. These alleles have been described as homozygous viable and male sterile [32], but in our hands, they were homozygous lethal but survived as adults when transheterozygous with *Df(3L)α1S1*. Subsequently, we examined the resulting transheterozygote mutant testes using immunofluorescence (Figure 2A–D). Compared to heterozygous *Df(3L)α1S1/+* controls, the morphological defects in each of *Df(3L)α1S1/Df(3L)α1S1*, *Df(3L)α1S1/Dα1^Z5234^* and *Df(3L)α1S1/Dα1^Z1703^* appeared similar. A loss of Vasa-positive early germ cells from around the somatic quiescent hub cells (labelled with an antibody to detect *Drosophila* E-cadherin) was evident in all genotypes, as was the presence of spermatocytes close to the apical hub (Figure 2B–D). Quantification of the number of Vasa-positive GSCs in direct contact with the hub in adults at seven days post-eclosion revealed a significant loss of GSCs from each of the mutant genotypes compared to *w^1118^* control testes. These results suggest an essential role for *Dα1* in the maintenance of *Drosophila* GSCs.

### 3.2. Expression of Dα1 in the Germline of Df(3L)α1S1/Df(3L)α1S1 Mutants Can Rescue GSC Loss, but Not Sterility

**Figure 3:**
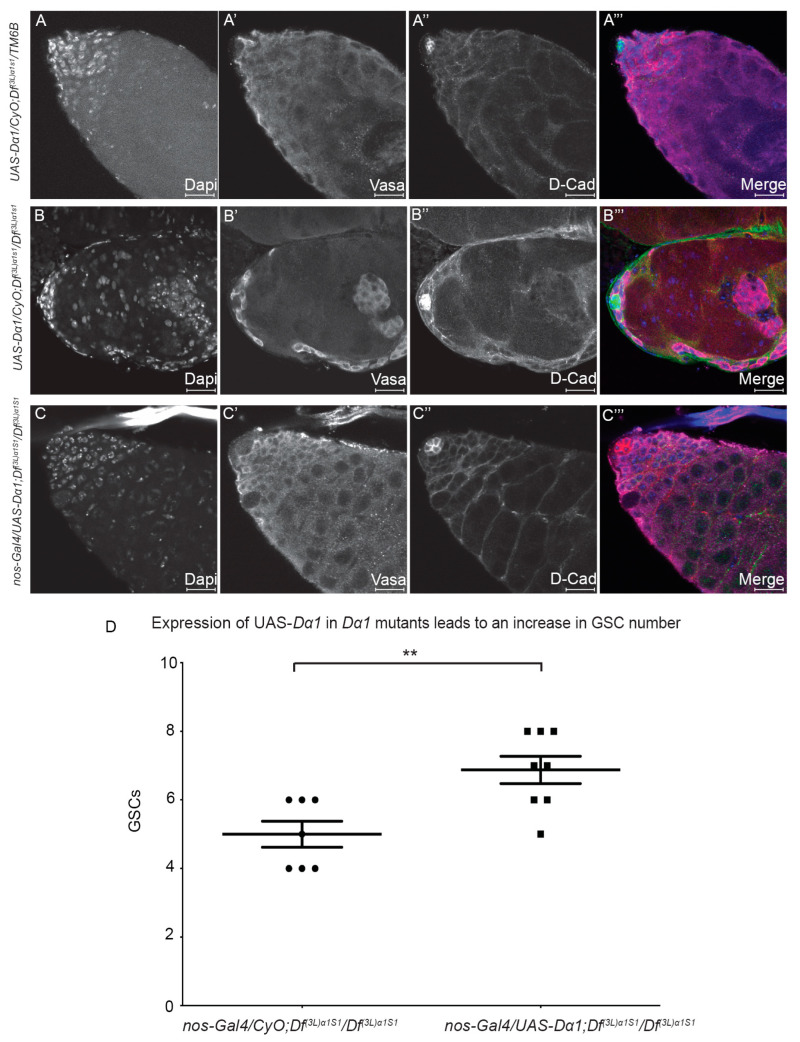
Expression of a *UAS-Dα1* transgene in early germline cells of *Dα1* homozygous deficiency mutant rescues germ cell loss. (**A**–**A’’’**) Representative confocal micrographs of testes dissected from newly eclosed *UAS-Dα1/CyO; Df(3L)α1S1/Tm6B* control; (**B**–**B’’’**) *UAS-Dα1/CyO; Df(3L)α1S1/Df(3L)α1S1* deficiency mutant and (**C**–**C’’’**) *nanos*-Gal4/*UAS-Dα1; Df(3L)α1S1/Df(3L)α1S1* flies. Labelling with Dapi (DNA), Vasa (germ cells) and D-Cad (hub and cyst cells) shows that loss of germ cells in the deficiency mutant (**B**–**B’’’**) could be rescued when *Dα1* is expressed in early germ cells via *nanos*-Gal4 in a deficiency background (**C**–**C’’’**); (**D**) Scatterplot and statistical analysis showing that the loss of GSCs in deficiency mutants [average of (5 (±1)) GSCs in newly eclosed animals] could be at least partially rescued when a *UAS-Dα1* transgene is expressed in early germ cells in the *Dα1* deficiency background [average of (6.87 (±1.13)) GSCs]. ** *p* < 0.01. Scale Bars, 20 μm.

Although we have shown the necessity for Dα1 for sustaining *Drosophila* GSCs through analysis of homozygous or transheterozygote flies, it remains unclear if its requirement is intrinsic to GSCs or if Dα1 is required within the surrounding somatic cells, which serve as the GSC niche. To further assess whether Dα1 is required in GSCs for their maintenance, we expressed a *UAS*-*Dα1* transgene in the germline of *Df(3L)α1S1/Df(3L)α1S1* mutants using a *nanos*-Gal4 driver, which selectively drives expression in GSCs and spermatogonial cells [33], and subsequently assayed for GSC rescue. Newly eclosed control *UAS-Dα1/CyO; Df(3L)α1S1/TM6B* heterozygous males were fertile, and immunofluorescence confirmed that testes displayed no obvious morphological abnormalities (Figure 3A–A’’’). In contrast, *UAS-Dα1/CyO; Df(3L)α1S1/Df(3L)α1S1* males in the absence of *nanos*-Gal4 were sterile as expected, and testes dissected from newly eclosed males exhibited a loss of early Vasa-positive germ cells from around the hub (Figure 3B–B’’’). After driving expression of *UAS-Dα1* with *nanos*-Gal4 in the *Dα1* deficiency background, testes dissected from newly eclosed *nos*-Gal4/*UAS-Dα1; Df(3L)α1S1/Df(3L)α1S1* exhibited no obvious morphological defects indicative of a rescue of early germ cells, including GSCs (Figure 3C–C’’’). Additionally, a significantly higher number of GSCs were observed in testes dissected from *nos*-Gal4/*UAS-Dα1; Df(3L)α1S1/Df(3L)α1S1* newly eclosed males compared to testes dissected from *UAS-Dα1/CyO; Df(3L)α1S1/Df(3L)α1S1* males at the same age point (Figure 3D), supporting the notion that Dα1 is required for the proper regulation and maintenance of GSCs. We mated at least nine individual *nos*-Gal4/*UAS-Dα1; Df(3L)α1S1/Df(3L)α1S1* males with 3–4 *w^1118^* females, and all remained sterile despite the rescue of early germ cells, which suggests a requirement for Dα1 at different stages or within multiple cell types throughout spermatogenesis. This observation is consistent with the pattern of *Dα1* mRNA, which is detected in early germ cells and more highly in spermatocytes (Supplementary Figure S1).

### 3.3. The Testis Phenotype of Df(3L)α1S1/Dα1^Z5234^ Mutants Is Affected by Temperature

**Figure 4:**
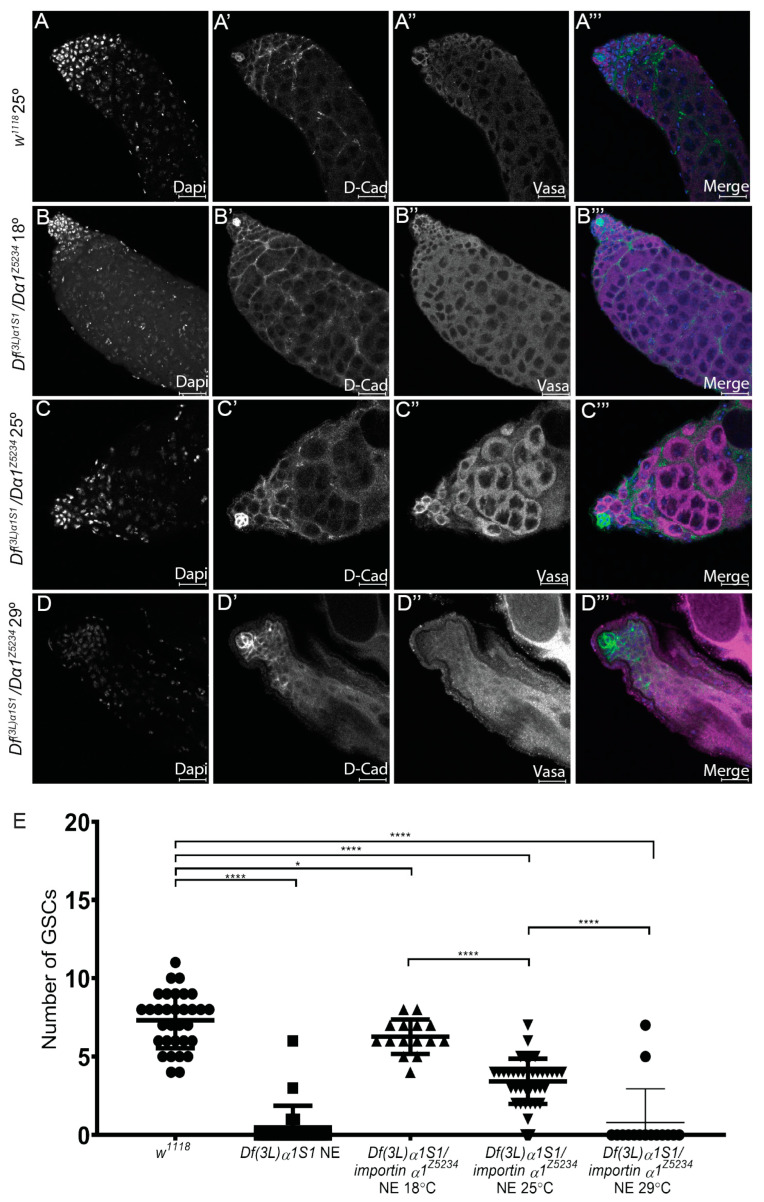
Temperature affects the severity of the *Df(3L)α1S1/Dα1^Z5234^* mutant phenotype. (**A**–**A’’’**) Representative confocal micrographs of testes dissected from *w^1118^* (control) testes raised at 25 °C; (**B**–**B’’’**) *Df(3L)α1S1/Dα1^Z5234^* transheterozygotes raised at 18 °C; (**C**–**C’’’**) 25 °C or (**D**–**D’’’**) 29 °C. Immunohistochemistry with a germ cell marker (Vasa) and a hub and cyst cell marker (D-Cad) shows a progression of the severity of loss of germ cells with increasing temperature. (**E**) Scatterplot with statistical analysis showing significant loss of GSCs when comparing adult *w^1118^* (control) testes to adult *Dα1* mutant testes. Testes dissected from *w^1118^* flies contained an average of 7.31 (±1.79) GSCs, which was significantly different from the number of GSCs counted in *Df(3L)α1S1* homozygotes (0.47 (±1.38)) GSCs, and *Df(3L)α1S1/Dα1^Z5234^* transheterozygotes raised at 18 °C (6.27 (±1.10)) GSCs. *Df(3L)α1S1/Dα1^Z5234^* transheterozygotes testes raised at 25 °C contained an average of 3.42 (±1.44) GSCs, which was significantly less than the number of GSCs counted in flies of the same genotype raised at 18 °C and 29° (0.80 (±2.14)) GSCs. * *p* < 0.05, **** *p* < 0.0001. Scale Bars, 20 μm.

Our analysis to date has shown a requirement for Dα1 in the maintenance of GSCs. Interestingly, quantification of GSC numbers in testes dissected from newly eclosed *Dα1* mutant adults (Figure 3D) and aged flies (Figure 2E) indicated that loss of Dα1 results in the progressive loss of GSCs over time. We questioned whether challenging *Dα1* mutants by increasing the temperature at which they were raised would affect the rate of GSC loss, as ezymatically driven developmental processes may be expected to be more rapid at higher temperatures, perhaps pointing to a function for Dα1 in regulating the rate of GSC division. To this end, we characterised (by immunofluorescence) testes dissected from newly eclosed *Df(3L)α1S1/Dα1^Z5234^* mutant flies raised at either 18 °C, 25 °C or 29 °C from the time of mating. Testes dissected from *w^1118^* (control) adult males reared at 25 °C and *Df(3L)α1S1/Dα1^Z5234^* mutants reared at 18 °C appeared similar in morphology (Figure 4A–B’’’), and mutant testes exhibited no obvious cellular defects (Figure 4B–B’’’). In contrast, testes dissected from *Df(3L)α1S1/Dα1^Z5234^* reared at either 25 °C or 29 °C exhibited a loss of Vasa-positive germ cells (Figure 4C–C’’’). Quantification of the number of GSCs in each of these samples confirmed that the severity of stem cell loss in *Df(3L)α1S1/Dα1^Z5234^* correlated with an increase in temperature (Figure 4E), with mutants reared at 29 °C exhibiting similar GSC numbers to testes dissected from newly eclosed *Df(3L)α1S1* homozygotes raised at 25 °C (Figure 4E). This is suggestive of the temperature-sensitive nature of *Dα1^Z5234,^* but these results also raise the possibility that other genes lost as a result of the deficiency could contribute to the loss of function phenotype in the homozygote deficiency strain.

### 3.4. Expression of a Dα1 Dominant Negative Transgene in GSCs Confirms a Functional Requirement for Dα1 in GSC Maintenance

**Figure 5:**
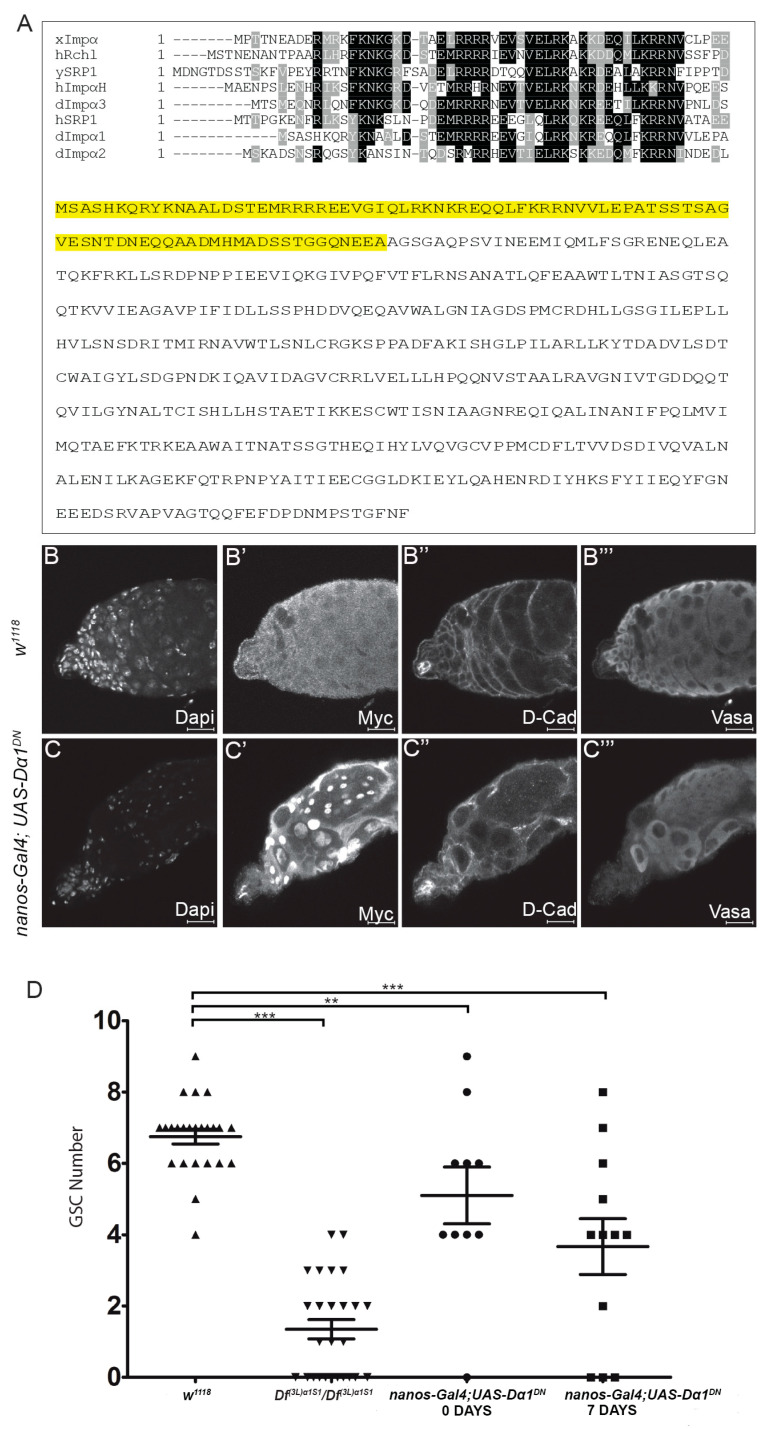
Expression of a dominant negative *Dα1* transgene driven in early germ cells results in a failure of stem cell maintenance. (**A**) Alignment of the Xenopus Importin α (isoform 1b) importin β binding domain against Human Rch1, Yeast SRP1, Human Importin α homologue, *Drosophila* Dα3, Human SRP1, *Drosophila* Dα1 and *Drosophila* Dα2. Full length amino acid sequence for Dα1 with the sequence removed in the creation of the dominant negative highlighted in yellow. Representative confocal micrographs of testes dissected from (**B**–**B’’’**) *w^1118^* and (**C**–**C’’’**) *nanos*-Gal4; *UAS-Dα1^DN^* adult males labelled with Dapi (DNA), Myc (*UAS-Dα1^DN^* is a Myc tagged transgene), D-Cad and Vasa. Testes dissected from *nanos*-Gal4; *UAS-Dα1^DN^* flies show a loss of early germ cells from around the somatic hub; (**D**) Scatterplot and statistical analysis showing that expression of the dominant negative transgene in early germ cells causes a significant loss of GSCs in the testes dissected from both newly eclosed adults [(5.10 ± (2.51)) GSCs] and aged adults [(7 days, 3.67 (± 2.71)) GSCs] when compared to the number of GSCs counted in testes dissected from control animals [*w^1118^* (6.75 ± (1.03)) GSCs]. The number of GSCs was counted in testes dissected from *Df(3L)α1S1* homozygotes as a reference (1.35 ± (1.38) GSCs). ** *p* < 0.01, *** *p* < 0.001. Scale Bars, 20 μm.

Whole-mutant analysis has revealed the importance of Dα1 in the regulation of GSC maintenance. To further determine the specificity of the role of Dα1 in germ cell maintenance, we generated a Dα1 dominant negative transgene (*UAS-Dα1^DN^*), which could be expressed in a cell-specific manner using the bipartite GAL4-UAS system. Based on sequence conservation of importin genes between *Drosophila melanogaster*, Xenopus, Yeast and Human, we generated a truncated copy of the *Dα1* coding sequence, which would result in the removal of the conserved importin β binding site [34,35,36] at the N terminus (Figure 5A). This construct was subsequently cloned into a pTMW Gateway vector with a 5x Myc tag and a pUASt promoter sequence. Driving expression of *UAS-Dα1^DN^* in GSCs using *nanos-Gal4* resulted in the loss of Vasa-positive GSCs from the somatic hub (Figure 5C–C’’’), a phenotype that was observed in *Df(3L)α1S1* homozygotes and *Df(3L)α1S1/Dα1^Z5234^* transheterozygotes. Quantification of the number of GSCs in testes dissected from *nanos-Gal4; UAS-Dα1^DN^* flies showed a significant loss of GSCs in both newly eclosed and aged adults (Figure 5D), and consistent with our previous results, we observed a progression of the severity of the phenotype with age. The persistence of Vasa-positive mature spermatocytes in the apex of the testis at 7 days post-eclosion (Figure 5C’’’) suggested to us that GSCs are progressively lost from the niche through premature differentiation rather than cell death and is consistent with the observation that in *Dα1* mutants, GSCs are lost over time.

### 3.5. Expression of a Dα1 Dominant Negative Transgene in Differentiated Germ Cells Does Not Affect Germ Cell Development/Differentiation

**Figure 6:**
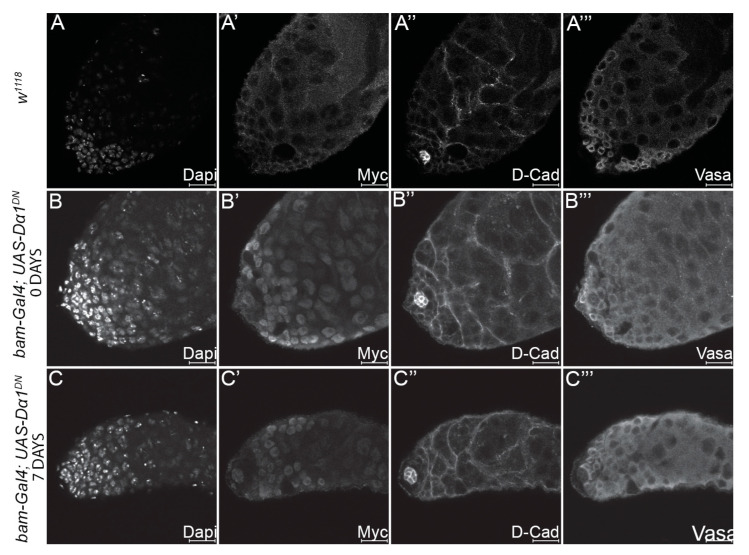
Expression of a dominant negative *Dα1* transgene in differentiated germ cells results in no visible defects in early spermatogenesis. Representative confocal micrographs of testes dissected from (**A**–**A’’’**) *w^1118^* control adults, and testes dissected from (**B**–**B’’’**) newly eclosed *bam-Gal4*; *UAS-Dα1^DN^* adults and (**C**–**C’’’**) aged adults. Labelling of testes with Dapi (DNA), Myc (to control for the presence of the tagged dominant negative transgene), D-Cad (hub and cyst cells) and Vasa (germ cells) reveals no obvious defects when the transgene is driven in the differentiated germ cells as opposed to GSCs. Scale Bars, 20 μm.

Our analysis has suggested that the loss of Dα1 from GSCs results in their premature differentiation. We hypothesised that if the Dα1 dominant negative transgene caused excess death in early germ cells, then driving the transgene in differentiated daughters should similarly lead to a loss of spermatogonial cells. To test this, we drove the dominant negative transgene specifically in differentiated germ cells using the *bam*-Gal4 driver. The native *bam* gene is expressed in spermatogonia, but *bam* is repressed in GSCs by BMP signalling [37]. Testes dissected from both newly eclosed and aged *bam-Gal4*; *UAS-Dα1^DN^* flies appeared morphologically similar to testes dissected from control flies when analysed by immunohistochemistry and confocal microscopy, with no obvious cellular defects (Figure 6A–C). These results support a requirement for Dα1 in GSC maintenance and prevention of premature differentiation of stem cells in the *Drosophila* testis.

## 4. Discussion

The original characterisation of *Df(3L)α1S1* reported that homozygotes were male-sterile and that dissected testes contained morphologically abnormal cysts of spermatocytes. This chromosomal deficiency deletes the coding sequence of six genes in addition to *Dα1*, but the phenotype was specifically associated with the loss of Dα1 function due to the ability of *Act5C-Gal4* and *UASp-Dα1* to rescue the fertility defect [28]. Phase contrast imaging of live testes and immunostaining of fixed tissue both allowed us to identify that Dα1 is also required for maintaining GSCs in the niche. We too observed abnormal spermatocyte cysts, and immunofluorescence studies using anti-Vasa to mark pre-meiotic germ cells enabled us to determine that, in addition to a loss of GSCs, the apical region of the testis contained cysts of spermatocytes (in positions where we would normally expect to find GSCs and spermatogonia) that contained variable numbers of cells, less than the normal number of 16 spermatocytes per cyst. This suggests to us that GSCs and spermatogonia are lost from the testis due to premature differentiation into spermatocytes. We also confirmed that the loss of early germ cells was specifically associated with the loss of Dα1 function, as transheterozygotes of two independent alleles of *Dα1* (containing premature termination codons) combined with *Df(3L)α1S1* recapitulated the phenotype observed in *Df(3L)α1S1* homozygotes.

We were also able to determine that the loss of GSCs and spermatogonia was due to a cell intrinsic requirement for Dα1, as we could rescue the germ cell loss via the expression of a *Dα1* transgene from a germ cell-specific promoter. Studies in mammalian cells have utilised dominant negative importin alpha alleles that have deletion of the importin-beta binding domain, which blocks importin alpha function by preventing it from transporting targets through the nuclear pore [34,35,36]. We generated an analogous transgene in *Drosophila*, which demonstrated a similar phenotype to the loss of *Dα1* alleles when driven by a germ cell-specific promoter. Moreover, this transgene displayed the loss of GSC and spermatogonia phenotype when combined with a Gal4 driver that expressed in GSCs and spermatogonia (*nanos-Gal4*) but not when combined with a driver that only expressed in the 4–16 cell stages of spermatogonia (*bam-Gal4*). These data imply that Dα1 is specifically required in GSCs (and potentially very early spermatogonial stages) and that a reduction in Dα1 function in maturing spermatogonia does not impact cell survival or differentiation of early germ cells. *Df(3L)α1S1* homozygotes exhibit degradation of spermatocytes (Supplementary Figure S1), suggesting that Dα1 is required in later germ cells, so it is also possible that the level of expression from *bam-Gal4* was not sufficient to induce a dominant negative phenotype.

We also found the phenotype associated with loss of Dα1 (in a truncation mutant in trans to the deficiency) to be temperature-sensitive, with a more severe phenotype associated with elevated temperature. The reason for this sensitivity is not clear at present, but it may be associated with increased cellular metabolism and cell turnover at higher temperatures. Interestingly, the loss of GSCs in *Df(3L)α1S1* at 25 °C was only equivalent to *Dα1^Z5234^/Df(3L)α1S1* at 29 °C, suggesting that although it has been reported that *Act5c-Gal4*-driven-Dα1 can rescue the fertility of *Df(3L)α1S1* homozygotes [28], one of the other six genes included within the deficiency may also play a role in GSC maintenance.

The Ratan (2008) study also showed that ectopic expression of Dα2 or Dα3 could not rescue infertility associated with *Df(3L)α1S1* homozygotes, implying a paralogue-specific role for importins in the testis. These data are consistent with importin alpha genes having different targets, and therefore specific importins are associated with specific cell biological and developmental roles. All three importin alpha genes are highly expressed in spermatocytes but appear not to be redundant, as loss of only Dα1 in *Df(3L)a1S1* (Supplementary Figure S1) results in these cells eventually degrading and not producing spermatids.

The importance of ensuring that the correct importin is expressed at the correct time can be demonstrated by a study that showed Dα2 must be excluded from *Drosophila* primordial germ cells (pole cells) for their development. A critical aspect of germ cell development is the prevention of inappropriate expression of somatic genes [38]. The RNA-binding protein Nanos has been shown to inhibit translation of maternally supplied Dα2 in pole cells, and this inhibition of Dα2 activity is required to prevent nuclear import of the Ftz-F1 transcription factor, which subsequently allows transcription of somatic genes if inappropriately expressed in the germline [39].

There may also be sex-specific requirements for importin alphas, as the Sex lethal mRNA-binding protein regulates Dα3 expression in *Drosophila* female primordial germ cells. Experiments that knocked down the combination of maternal and zygotic Dα3 activity in embryos demonstrated a subsequent decrease in the number of GSCs present in adults in ovaries, with some ovaries being devoid of germ cells, but no apparent effect in testes [40].

Although Dα1 mutants have been reported as viable but infertile, they do have other phenotypes. Dα1 plays an important role in setting the circadian clock within specific neurons via regulating the nuclear entry of the Period and Timeless clock proteins. Flies that have Dα1 knocked down within clock neurons exhibit arrhythmic behaviour [41], demonstrating that Dα1 has a unique set of functional requirements within specific tissues.

A greater understanding of importin function will not only facilitate our understanding of developmental processes but may uncover an important mechanism to combat disease phenotypes. Pathological variants of the Tau protein have been linked to Alzheimer’s disease, and, more broadly, are associated with a group of neurodegenerative diseases known as tauopathies. A study that utilised pharmacological inhibitors of importin activity was able to prevent nuclear importation of pathological Tau proteins and inhibit the cell death that is associated with their expression [42]. In a similar manner, genetic experiments in *Drosophila* have shown that Dα3 is required for the transportation of a pathogenic truncated Ataxin-3 variant protein into nuclei, which then results in neuronal toxicity [43].

## 5. Conclusions

This study has demonstrated a role for Dα1 in maintaining the germline stem cell pool within the *Drosophila* testis niche. We do not yet know what proteins are imported via a Dα1-specific mechanism that is critical for GSC function. This will clearly be important to understand why Dα1 has a specific requirement in male GSCs. Future studies are also needed to determine if Dα1 is required in other stem cell populations within adult *Drosophila* or if paralogous importin alphas have specific roles in stem cells associated with other tissues.

## Data Availability

The original contributions presented in the study are included in the article/Supplementary Materials, further inquiries can be directed to the corresponding author.

## Supplementary Materials

The following supporting information can be downloaded at: https://www.mdpi.com/article/10.3390/cells13060494/s1, Figure S1: Dα1 protein and mRNA expression in the testis. Reference [44] is cited in Supplementary Materials.

## References

[1] Lu J., Wu T., Zhang B., Liu S., Song W., Qiao J., Ruan H. (2021). Types of nuclear localization signals and mechanisms of protein import into the nucleus. Cell Commun. Signal..

[2] Nathaniel B., Whiley P.A.F., Miyamoto Y., Loveland K.L. (2022). Importins: Diverse roles in male fertility. Semin. Cell Dev. Biol..

[3] Wing C.E., Fung H.Y.J., Chook Y.M. (2022). Karyopherin-mediated nucleocytoplasmic transport. Nat. Rev. Mol. Cell Biol..

[4] Mosammaparast N., Pemberton L.F. (2004). Karyopherins: From nuclear-transport mediators to nuclear-function regulators. Trends Cell Biol..

[5] Macara I.G. (2001). Transport into and out of the nucleus. Microbiol. Mol. Biol. Rev..

[6] Goldfarb D.S., Corbett A.H., Mason D.A., Harreman M.T., Adam S.A. (2004). Importin alpha: A multipurpose nuclear-transport receptor. Trends Cell Biol..

[7] Stewart M. (2007). Molecular mechanism of the nuclear protein import cycle. Nat. Rev. Mol. Cell Biol..

[8] Chook Y.M., Suel K.E. (2011). Nuclear import by karyopherin-betas: Recognition and inhibition. Biochim. Biophys. Acta.

[9] Kimura M., Morinaka Y., Imai K., Kose S., Horton P., Imamoto N. (2017). Extensive cargo identification reveals distinct biological roles of the 12 importin pathways. eLife.

[10] Prieve M.G., Guttridge K.L., Munguia J.E., Waterman M.L. (1996). The nuclear localization signal of lymphoid enhancer factor-1 is recognized by two differentially expressed Srp1-nuclear localization sequence receptor proteins. J. Biol. Chem..

[11] Kohler M., Ansieau S., Prehn S., Leutz A., Haller H., Hartmann E. (1997). Cloning of two novel human importin-alpha subunits and analysis of the expression pattern of the importin-alpha protein family. FEBS Lett..

[12] Tsuji L., Takumi T., Imamoto N., Yoneda Y. (1997). Identification of novel homologues of mouse importin alpha, the alpha subunit of the nuclear pore-targeting complex, and their tissue-specific expression. FEBS Lett..

[13] Gorlich D., Henklein P., Laskey R.A., Hartmann E. (1996). A 41 amino acid motif in importin-alpha confers binding to importin-beta and hence transit into the nucleus. EMBO J..

[14] Weis K., Ryder U., Lamond A.I. (1996). The conserved amino-terminal domain of hSRP1 alpha is essential for nuclear protein import. EMBO J..

[15] Conti E., Uy M., Leighton L., Blobel G., Kuriyan J. (1998). Crystallographic analysis of the recognition of a nuclear localization signal by the nuclear import factor karyopherin alpha. Cell.

[16] Dockendorff T.C., Tang Z., Jongens T.A. (1999). Cloning of karyopherin-alpha3 from *Drosophila* through its interaction with the nuclear localization sequence of germ cell-less protein. Biol. Chem..

[17] Kussel P., Frasch M. (1995). Pendulin, a *Drosophila* protein with cell cycle-dependent nuclear localization, is required for normal cell proliferation. J. Cell Biol..

[18] Torok I., Strand D., Schmitt R., Tick G., Torok T., Kiss I., Mechler B.M. (1995). The overgrown hematopoietic organs-31 tumor suppressor gene of *Drosophila* encodes an Importin-like protein accumulating in the nucleus at the onset of mitosis. J. Cell Biol..

[19] Fang X., Chen T., Tran K., Parker C.S. (2001). Developmental regulation of the heat shock response by nuclear transport factor karyopherin-alpha3. Development.

[20] Giarre M., Torok I., Schmitt R., Gorjanacz M., Kiss I., Mechler B.M. (2002). Patterns of importin-alpha expression during *Drosophila* spermatogenesis. J. Struct. Biol..

[21] Hogarth C.A., Calanni S., Jans D.A., Loveland K.L. (2006). Importin alpha mRNAs have distinct expression profiles during spermatogenesis. Dev. Dyn..

[22] Whiley P.A., Miyamoto Y., McLachlan R.I., Jans D.A., Loveland K.L. (2012). Changing subcellular localization of nuclear transport factors during human spermatogenesis. Int. J. Androl..

[23] Miyamoto Y., Sasaki M., Miyata H., Monobe Y., Nagai M., Otani M., Whiley P.A.F., Morohoshi A., Oki S., Matsuda J. (2020). Genetic loss of importin alpha4 causes abnormal sperm morphology and impacts on male fertility in mouse. FASEB J..

[24] Mason D.A., Fleming R.J., Goldfarb D.S. (2002). Drosophila melanogaster importin alpha1 and alpha3 can replace importin alpha2 during spermatogenesis but not oogenesis. Genetics.

[25] Mathe E., Bates H., Huikeshoven H., Deak P., Glover D.M., Cotterill S. (2000). Importin-alpha3 is required at multiple stages of Drosophila development and has a role in the completion of oogenesis. Dev. Biol..

[26] Gorjanacz M., Adam G., Torok I., Mechler B.M., Szlanka T., Kiss I. (2002). Importin-alpha 2 is critically required for the assembly of ring canals during *Drosophila* oogenesis. Dev. Biol..

[27] Mihalas B.P., Western P.S., Loveland K.L., McLaughlin E.A., Holt J.E. (2015). Changing expression and subcellular distribution of karyopherins during murine oogenesis. Reproduction.

[28] Ratan R., Mason D.A., Sinnot B., Goldfarb D.S., Fleming R.J. (2008). *Drosophila* importin alpha1 performs paralog-specific functions essential for gametogenesis. Genetics.

[29] Fabian L., Brill J.A. (2012). *Drosophila* spermiogenesis: Big things come from little packages. Spermatogenesis.

[30] Siddall N.A., Hime G.R. (2017). A *Drosophila* toolkit for defining gene function in spermatogenesis. Reproduction.

[31] Koundakjian E.J., Cowan D.M., Hardy R.W., Becker A.H. (2004). The Zuker collection: A resource for the analysis of autosomal gene function in *Drosophila* melanogaster. Genetics.

[32] Lindsley D.L., Roote J., Kennison J.A. (2013). Anent the genomics of spermatogenesis in *Drosophila* melanogaster. PLoS ONE.

[33] Van Doren M., Williamson A.L., Lehmann R. (1998). Regulation of zygotic gene expression in *Drosophila* primordial germ cells. Curr. Biol..

[34] Aguilar A., Wagstaff K.M., Suarez-Sanchez R., Zinker S., Jans D.A., Cisneros B. (2015). Nuclear localization of the dystrophin-associated protein alpha-dystrobrevin through importin alpha2/beta1 is critical for interaction with the nuclear lamina/maintenance of nuclear integrity. FASEB J..

[35] Sekimoto T., Imamoto N., Nakajima K., Hirano T., Yoneda Y. (1997). Extracellular signal-dependent nuclear import of Stat1 is mediated by nuclear pore-targeting complex formation with NPI-1, but not Rch1. EMBO J..

[36] Ushijima R., Sakaguchi N., Kano A., Maruyama A., Miyamoto Y., Sekimoto T., Yoneda Y., Ogino K., Tachibana T. (2005). Extracellular signal-dependent nuclear import of STAT3 is mediated by various importin alphas. Biochem. Biophys. Res. Commun..

[37] Kawase E., Wong M.D., Ding B.C., Xie T. (2004). Gbb/Bmp signaling is essential for maintaining germline stem cells and for repressing bam transcription in the *Drosophila* testis. Development.

[38] Hayashi Y., Hayashi M., Kobayashi S. (2004). Nanos suppresses somatic cell fate in Drosophila germ line. Proc. Natl. Acad. Sci. USA.

[39] Asaoka M., Hanyu-Nakamura K., Nakamura A., Kobayashi S. (2019). Maternal Nanos inhibits Importin-alpha2/Pendulin-dependent nuclear import to prevent somatic gene expression in the *Drosophila* germline. PLoS Genet..

[40] Ota R., Morita S., Sato M., Shigenobu S., Hayashi M., Kobayashi S. (2017). Transcripts immunoprecipitated with Sxl protein in primordial germ cells of *Drosophila* embryos. Dev. Growth Differ..

[41] Jang A.R., Moravcevic K., Saez L., Young M.W., Sehgal A. (2015). Drosophila TIM binds importin alpha1, and acts as an adapter to transport PER to the nucleus. PLoS Genet..

[42] Candia R.F., Cohen L.S., Morozova V., Corbo C., Alonso A.D. (2022). Importin-Mediated Pathological Tau Nuclear Translocation Causes Disruption of the Nuclear Lamina, TDP-43 Mislocalization and Cell Death. Front. Mol. Neurosci..

[43] Cho J.H., Jo M.G., Kim E.S., Lee N.Y., Kim S.H., Chung C.G., Park J.H., Lee S.B. (2022). CBP-Mediated Acetylation of Importin alpha Mediates Calcium-Dependent Nucleocytoplasmic Transport of Selective Proteins in *Drosophila* Neurons. Mol. Cells.

[44] Li H., Janssens J., De Waegeneer M., Kolluru S.S., Davie K., Gardeux V., Saelens W., David F.P.A., Brbic M., Spanier K. (2022). Fly Cell Atlas: A single-nucleus transcriptomic atlas of the adult fruit fly. Science.

