# Supplementary material for: Drosophila Importin Alpha 1 (Dα1) Is Required to Maintain Germline Stem Cells in the Testis Niche
Source: Cells. 2024 Mar 12;13(6):494. doi: 10.3390/cells13060494 (PMC10969130; doi:10.3390/cells13060494)
Supplement: Supplementary file 1 [file cells-13-00494-s001.zip › cells-2880144-SI.pdf]

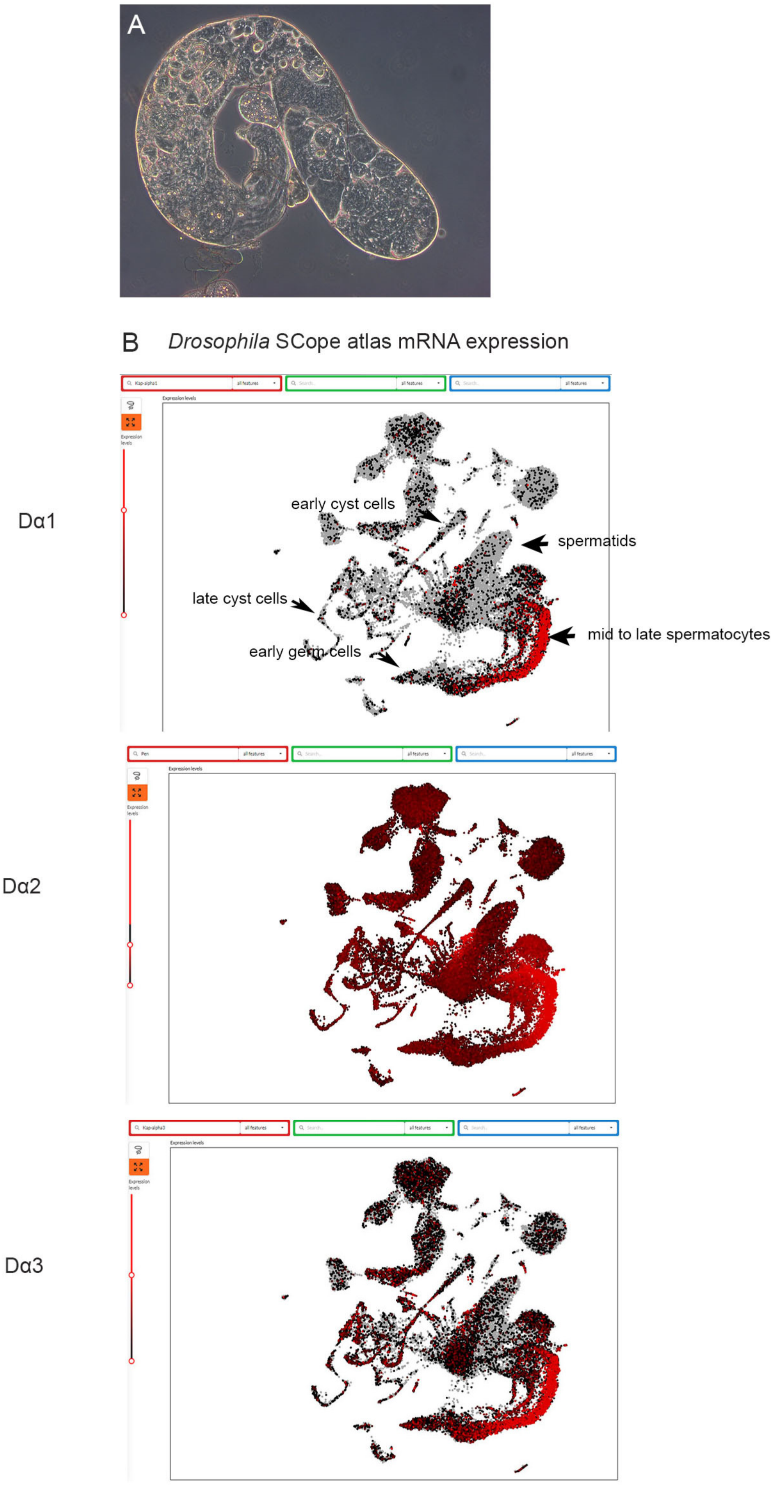

Supplementary Figure S1. *Df(3L)α1S1* phenotype and mRNA expression of *importin alpha* genes in the testis.
(**A**) *Df(3L)α1S1* spermatocytes do not progress to viable spermatids; (**B**) *Dα1*, *Dα2* and *Dα3* mRNA expression as detected using the Fly Cell Atlas resource SCope [44]. All three genes are expressed throughout germ cell development with highest levels of expression in spermatocytes. Default FCA UMAP settings are shown with black illustrating lower expression and red high - level expression.
